# Comment on Castellucci et al. Impaired Vestibulo-Ocular Reflex on Video Head Impulse Test in Superior Canal Dehiscence: “Spontaneous Plugging” or Endolymphatic Flow Dissipation? *Audiol. Res.* 2023, *13*, 802–820

**DOI:** 10.3390/audiolres14050072

**Published:** 2024-09-23

**Authors:** Eugen Constant Ionescu, Eugenia Mustea, Pierre Reynard, Hung Thai-Van

**Affiliations:** 1 Department of Audiology and Neurotology, Hospices Civils de Lyon, 69003 Lyon, France; eugenia.mustea@chu-lyon.fr (E.M.); pierre.reynard@chu-lyon.fr (P.R.); hung.thai-van@chu-lyon.fr (H.T.-V.); 2 Paris Hearing Institute, Institut Pasteur, Inserm U1120, 75015 Paris, France; 3 Department of Physiology, Claude Bernard Lyon 1 University, 69003 Lyon, France

We read, with great interest, two recent articles by Castellucci and al. [1,2] published in Audiology Research. The author has taken a close interest over time in the phenomenon of spontaneous self-plugging in patients with superior semicircular canal dehiscence (SSCD) and, more recently, in the case of posterior semicircular canal dehiscence (PSCD) [3]. Even if the pathology of the third mobile window (TMW) described in its first anatomical variant by Minor et al. in 1998 [4] has raised a great interest in research for several years, there is still a significant lack of knowledge related to its origin, pathophysiology and especially its natural evolution. That is why, in this context, the hypothesis put forward by Castelucci et al. in the articles cited above, according to which the micromechanics of the endolymphatic fluid in the case of spontaneous auto-plugging of the SSCD would be disturbed by decreasing the flow to the ampulla of the semicircular canal (SC), is interesting and plausible in our opinion.

However, we think that it cannot be assumed that this could be a general rule for any natural evolution in otic capsule dehiscence (OCD), especially in the case of SSCD. As an example, in a case report we previously published, also cited by Dr Castelucci, a patient presented with a partial auto-plugging of the left SSCD (Figure 1) at the origin of an otolith’s entrapment towards the ampullary part of the respective SC [5]. A persistent left SSC BPPV was observed despite multiple repositioning maneuvers. Although the patient underwent a rigorous follow-up, the Video Head Impulse Test’s (VHIT) gain of the left superior SC remains surprisingly normal for a long period (Figure 1C).

We should add here, in support, a case of a spontaneous and complete SSCD auto-plugging confirmed by 3D labyrinthine MRI; that would also be the very first case, to the best of our knowledge, that had been reported and proven by dedicated imagery (Figure 2A,B). This professional musician patient was seriously embarrassed, especially by the persistent and significant right autophony apart from the slight and permanent dizziness. Apart from the imaging showing a complete plugging of the superior SC, its respective gain assessed with VHIT (Synapsis, Marseille, France) (Figure 2C) was found to be strictly normal. This apparent contradictory finding is nevertheless consistent with the data from the literature in the case of SC hypoplasia or aplasia that have frequently reported normal gains with VHIT in this type of vestibular abnormalities [6]. This fact suggests that even in the case of a minimal available column of endolymph to be mobilized towards a functional SC’s ampulla, we can expect a normal or quasi-normal function according to Ewald’s laws [7] for high frequency vestibular stimulations. Thus, the question can only be raised as to what extent the rapidity with which the auto-plugging process is established as correlated or not with an eventual secondary deficit of the Vestibulo-Ocular Reflex, which is measurable with various available VHIT systems as reported by some authors [8]. In addition, the atypical audiometric results for a symptomatic TMW presented here (Figure 1D and Figure 2D) as in some of the cases reported by Castellucci et al. should sensitize ENT specialists to the fact that certain large SSCD can progressively evolve with minimal audiological symptoms and signs. Moreover, as we have shown recently, even multiple otic capsule dehiscence can evolve with mixed and/or sensorineural hearing loss thus encountering the risk to be investigated only with MRI and not with a CT scan of the petrous bone, which can lead to misdiagnosis [9]. On the other hand, the merit of the last paper of Castellucci et al. consists in the fact that it sheds light on the probability that the VHIT’s gain in this variant (PSCD) to be diminished compared to SSCD is higher, and this is due to the anatomical conditions at the level of the posterior fossa. A limitation regarding the sensitivity of VHIT in the case of suspected PSCD would appear in older patients in whom presbyvestibulopathy phenomena predominate [10], which would thus hypothetically decrease the specificity of this test to identify a possible auto-plugging process.

In any case, all these observations and new hypotheses regarding the clinical “atypia” reported in symptomatic patients in relation to a known variant of OCD must motivate medical research, since we still have a lot to learn about this before mimicking and consequently challenging pathology.

## Figures and Tables

**Figure 1:**
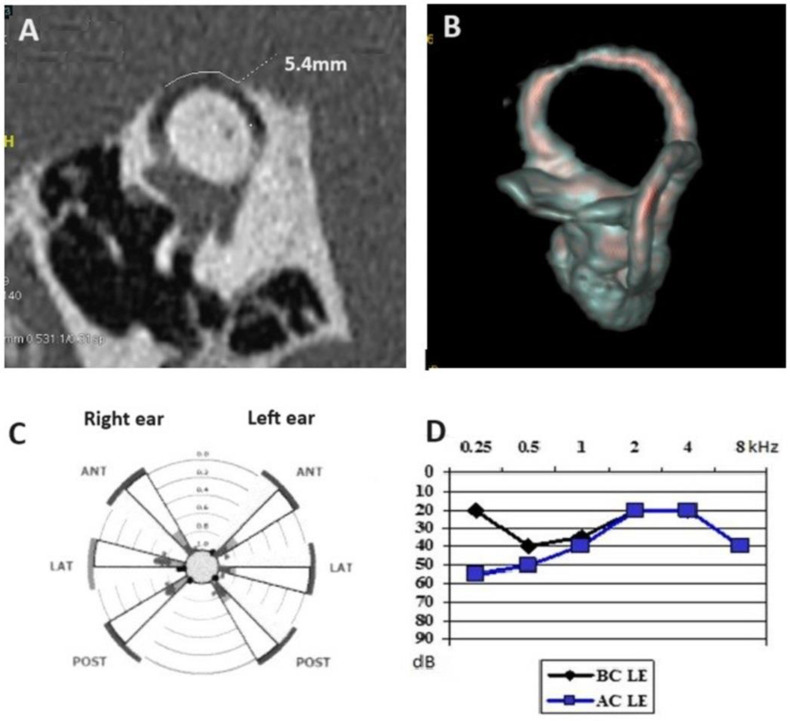
(**A**) High-resolution computed tomography (HRCT) scan of the petrous bone showing a 5.4 mm left-sided large dehiscence of the SSC—Pöschl incidence. (**B**) 3D HR MRI of the labyrinth confirming a narrowing at 0.5 mm of the SSC (for a normal diameter of about 1.2 mm)—multiaxial 3D plane view; (**C**) Video Head Impulse test showing normal gains for all SCC. (**D**) Left-sided mixed hearing loss with an air-bone gap of 40 dB at 0.25 kHz. SSC, superior semicircular canal. 3D HR MRI, three-dimensional high-resolution magnetic resonance imaging.

**Figure 2:**
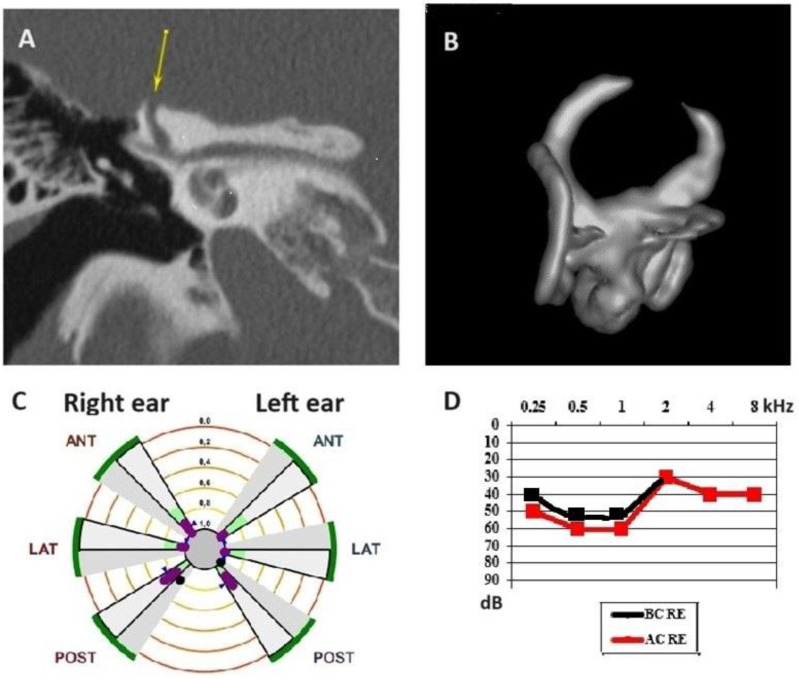
(**A**) High-resolution computed tomography (HRCT) scan of the petrous bone showing a right-sided large dehiscence of the SSC coronal incidence (yellow arrow). (**B**) 3D HR MRI of the labyrinth confirming spontaneous and complete SSCD auto-plugging; multiaxial 3D plane view; (**C**) Video Head Impulse test showing normal gains for all SCC. (**D**) Right-sided hearing loss. SSC, superior semicircular canal. 3D HR MRI, three-dimensional high-resolution magnetic resonance imaging.

## References

[1] Castellucci A., Malara P., Martellucci S., Alfarghal M., Brandolini C., Piras G., Armato E., Ruberto R.R., Brizzi P., Presutti L. (2023). Impaired Vestibulo-Ocular Reflex on Video Head Impulse Test in Superior Canal Dehiscence: “Spontaneous Plugging” or Endolymphatic Flow Dissipation?. Audiol. Res..

[2] Castellucci A., Dumas G., Abuzaid S.M., Armato E., Martellucci S., Malara P., Alfarghal M., Ruberto R.R., Brizzi P., Ghidini A. (2024). Posterior Semicircular Canal Dehiscence with Vestibulo-Ocular Reflex Reduction for the Affected Canal at the Video-Head Impulse Test: Considerations to Pathomechanisms. Audiol. Res..

[3] Castellucci A., Brandolini C., Piras G., Del Vecchio V., Modugno G.C., Ghidini A., Pirodda A. (2018). Spontaneous plugging of superior canal: Two possible natural evolutions of an “unstable” dehiscence. J. Vestib. Res..

[4] Minor L.B., Solomon D., Zinreich J.S., Zee D.S. (1998). Sound- and/or pressure-induced vertigo due to bone dehiscence of the superior semicircular canal. Arch. Otolaryngol. Head. Neck Surg..

[5] Ionescu E.C., Idriss S., Reynard P., Ltaief-Boudrigua A., Thai-Van H. (2022). Persistent Positional Vertigo in a Patient with Partial “Auto-Plugged” Superior Semicircular Canal Dehiscence: A Case Study. J. Int. Adv. Otol..

[6] Yun J.M., Kim S.H., Bae S.H. (2024). Vestibular dysfunction in lateral semicircular canal dysplasia. Front. Neurol..

[7] Ewald J.R. (1892). Physiologische Untersuchungen über das Endorgan des Nervus Octavus.

[8] Lee S.Y., Bae Y.J., Kim M., Song J.J., Choi B.Y., Koo J.W. (2020). Changes in Vestibulo-Ocular Reflex Gain after Surgical Plugging of Superior Semicircular Canal Dehiscence. Front. Neurol..

[9] Ionescu E.C., Reynard P., Damien M., Ltaief-Boudrigua A., Hermann R., Gianoli G.J., Thai-Van H. (2023). Why should multiple dehiscences of the otic capsule be considered before surgically treating patients with superior semicircular canal dehiscence? A radiological monocentric review and a case series. Front. Neurol..

[10] Agrawal Y., Van de Berg R., Wuyts F., Walther L., Magnusson M., Oh E., Sharpe M., Strupp M. (2019). Presbyvestibulopathy: Diagnostic criteria Consensus document of the classification committee of the Bárány Society. J. Vestib. Res..

